# *QuickStats:* Rates* of Death Due to Unintentional Injury from Fire or Flames,^†^ by Sex and Urbanization Level^§^ — National Vital Statistics System, United States, 2021

**DOI:** 10.15585/mmwr.mm7214a5

**Published:** 2023-04-07

**Figure:**
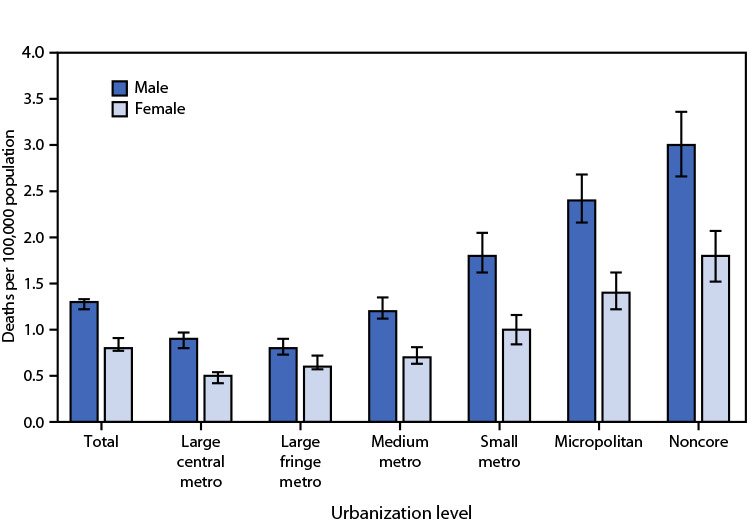
In 2021, the rates of death due to unintentional injury from fire or flames were 1.3 per 100,000 population among males and 0.8 among females and were higher for males than for females at each level of urbanization. Rates among males were lowest in large fringe (0.8) and large central (0.9) metropolitan areas and then increased with decreasing urbanization to 3.0 in noncore areas. Rates among females were lowest in large central metropolitan areas (0.5) and increased with decreasing urbanization to 1.8 in noncore areas.

**Source:** National Center for Health Statistics, National Vital Statistics System, Mortality Data, 2021. https://www.cdc.gov/nchs/nvss/deaths.htm

